# Brain morphology of the threespine stickleback (*Gasterosteus aculeatus*) varies inconsistently with respect to habitat complexity: A test of the Clever Foraging Hypothesis

**DOI:** 10.1002/ece3.2918

**Published:** 2017-04-04

**Authors:** Newaz I. Ahmed, Cole Thompson, Daniel I. Bolnick, Yoel E. Stuart

**Affiliations:** ^1^ Department of Integrative BiologyUniversity of Texas at AustinAustinTXUSA; ^2^ Department of Internal MedicineUniversity of Texas‐SouthwesternDallasTXUSA

**Keywords:** Clever Foraging Hypothesis, habitat complexity, neuroanatomy, spatial learning, teleost

## Abstract

The Clever Foraging Hypothesis asserts that organisms living in a more spatially complex environment will have a greater neurological capacity for cognitive processes related to spatial memory, navigation, and foraging. Because the telencephalon is often associated with spatial memory and navigation tasks, this hypothesis predicts a positive association between telencephalon size and environmental complexity. The association between habitat complexity and brain size has been supported by comparative studies across multiple species but has not been widely studied at the within‐species level. We tested for covariation between environmental complexity and neuroanatomy of threespine stickleback (*Gasterosteus aculeatus*) collected from 15 pairs of lakes and their parapatric streams on Vancouver Island. In most pairs, neuroanatomy differed between the adjoining lake and stream populations. However, the magnitude and direction of this difference were inconsistent between watersheds and did not covary strongly with measures of within‐site environmental heterogeneity. Overall, we find weak support for the Clever Foraging Hypothesis in our study.

## 1 Introduction

The Clever Foraging Hypothesis (CFH) posits that organisms living in more complex environments will require greater neurobiological capacity to navigate and forage for food (Park & Bell, 2010; Parker & Gibson, 1977). Tasks that are more important tend to be managed by brain regions that are comparatively larger (Dukas, 1999), and thus, the CFH also predicts that organisms in more complex environments should have larger brains, all else equal (Kotrschal et al., 1998).

The CFH predicts, in particular, that more complex environments will require larger telencephala because the telencephalon is involved in higher level cognition and spatial memory crucial for foraging (Bauchot et al., 1977; Broglio et al., 2003, 2005; Burns & Rodd, 2008; Burns et al., 2009; Corfield et al., 2012; Gonda et al., 2009; Huntigford & Wright, 1989; Huntingford & Wright, 1992; Kotrschal et al., 1998; Park & Bell, 2010; Pollen et al., 2007; Powell & Leal, 2012; Rodriguez et al., 2002; Sherry, 2006; Timmermans et al., 2000; Warburton, 1990). This correlation between larger telencephala and higher level cognition has been documented for a variety of taxa, including birds (Corfield et al., 2012; Timmermans et al., 2000), reptiles (Powell & Leal, 2012; Rodriguez et al., 2002), and fish (Burns & Rodd, 2008; Burns et al., 2009; Gonda et al., 2009; Huntigford & Wright, 1989, 1992; Pollen et al., 2007; Rodriguez et al., 2002). Of course, other brain regions play a role in foraging and might also vary in size accordingly. Fish species living in complex environments have not only relatively larger telencephala (Bauchot et al., 1977; Broglio et al., 2003, 2005; Gonda et al., 2009; Park & Bell, 2010; Rodriguez et al., 2002), but also relatively larger cerebella (Kotrschal et al., 1998), larger occipital lobes for chemosensation (Bauchot et al., 1977; Kotrschal et al., 1998), and larger brains overall (Huntigford & Wright, 1992; Kotrschal et al., 1998; Park & Bell, 2010).

These correlative, comparative studies suggest that habitat complexity drives brain size evolution, but few studies have investigated brain differences among conspecific populations inhabiting contrasting environments to test the CFH (see; Burns & Rodd, 2008; Burns et al., 2009; Gonda et al., 2009; Park & Bell, 2010 for exceptions), thereby making it unclear whether interspecific findings are supported by intraspecific patterns. Furthermore, many studies of the brain–habitat relationship do not quantitatively measure environmental complexity, relying instead on qualitative descriptions of habitat type or use (e.g., Bauchot et al., 1977; Corfield et al., 2012; Powell & Leal, 2012). Here, we report a test of the CFH that incorporates quantitative estimates of complexity.

The threespine stickleback (*Gasterosteus aculeatus)*, which inhabits freshwater habitats throughout the Nearctic and Palaearctic, provides a powerful system to remedy this knowledge gap. Stickleback have repeatedly evolved phenotypic and genetic differences between populations inhabiting adjoining lake and stream environments (Hirase et al., 2014; McGee et al., 2013; McPhail, 1985, 1993; Odling‐Smee et al., 2008; Taylor & McPhail, 1999, 2000), thereby allowing us to test the CFH across discrete habitat types, similar to most studies of the CFH. However, because lakes and streams vary widely from one another in their environmental characteristics (Stuart et al., *in revision*), we can go a step further to test the CFH for the predicted correlation between neuroanatomy and quantitative measures of environmental complexity.

To our knowledge, only one study has investigated threespine stickleback brain morphology with respect to environmental complexity across contrasting habitats (Park & Bell, 2010; though see Gonda et al., 2009 for ninespine stickleback, *Pungitius pungitius*). Park and Bell (2010) compared shallow and deep lake populations of threespine stickleback, assuming that shallow lakes with more benthic habitat had greater habitat complexity than deep lakes with more open‐water, limnetic habitat. Counter to CFH expectations, stickleback from shallow lakes had smaller telencephala relative to deep lakes. Those telencephala were more laterally convex, however, perhaps indicative of greater neurological capacity (Park & Bell, 2010), but Park and Bell also suggest that simply categorizing lakes as either limnetic or benthic might be insufficient to capture the complexity of habitats where fish actually forage.

Here, we report a test of the CFH in threespine stickleback (*Gasterosteus aculeatus*) from 30 populations: 15 lake–stream population pairs, each pair from a different watershed, on Vancouver Island, British Columbia. We test the CFH by quantifying multivariate habitat complexity at each site, predicting that more complex habitats are correlated with larger brain (and brain subregion) size. We also ask whether there are consistent, “parallel” differences between lake and stream stickleback in brain morphology.

## 2 Materials and methods

### 2.1 Sample collection

In May‐June 2013, we collected adult threespine stickleback (*Gasterosteus aculeatus*) from sites in 15 lakes and their respective adjoining outlet streams (30 sites in total, Table 1). We chose lake–stream pairs from independent watersheds to minimize the influence of between‐pair migration and shared evolutionary history, and confirmed the independence of these replicate lake–stream pairs with genomic analyses of SNPs generated via ddRAD‐seq (Stuart et al., *in revision*). To capture fish, at each site we set a transect of 50 unbaited minnow traps, deployed haphazardly along a ~100 m stretch of lake shoreline or stream length. Specimens were euthanized with neutral buffered Tricaine (MS‐222) and stored in formalin, which does not appreciably affect brain size (Schander & Halanych, 2003). At the Universit of Texas at Austin, the fish were stained with Alizarin Red following standard protocols then stored in 40% isopropanol. Approximately 1 year passed between initial storage in isopropanol and examination of brain structure. Collections were conducted with approval from the British Columbia Ministry of Land, Environment, and Natural Resources (NA12‐84189 and NA13‐85697), and from the University of Texas at Austin Institutional Animal Use and Care Committee (AUP‐2012‐00065 and AUP‐2014‐00293).

**Table 1:** Collection localities and sample sizes. Latitude and longitude reported in UTM units

Lake–Stream pair	Habitat	Latitude	Latitude	Sample size	Stream type
Beaver (Be)	Lake	09U 0619582	5606817	18	–
	Stream	09U 0616749	5605855	17	Outlet
Boot (Bo)	Lake	10U 0318369	5548456	18	–
	Stream	10U 0316648	5546666	15	Outlet
Comida (Co)	Lake	10U 0319414	5558451	18	–
	Stream	10U 0319791	5556582	20	Outlet
Frederick (Fr)	Lake	10U 0351596	5413659	23	–
	Stream	10U 0353269	5416290	23	Outlet
Joe's (Jo)	Lake	09U 0607251	5609043	23	–
	Stream	09U 0604727	5609143	23	Outlet
Kennedy (Ke)	Lake	10U 0311216	5441254	24	–
	Stream	10U 0309736	5441838	21	Outlet
Moore (Mo)	Lake	09U 0564961	5601511	18	–
	Stream	09U 0564762	5602364	20	Outlet
Muchalat (Mu)	Lake	09U 0703713	5528557	20	–
	Stream	09U 0705063	5527674	21	Outlet
Northy (No)	Lake	10U 0344515	5520778	21	–
	Stream	10U 0345063	5520248	20	Outlet
Pachena (Pa)	Lake	10U 0350871	5411808	15	–
	Stream	10U 0349012	5410362	22	Outlet
Pye (Py)	Lake^a^	10U 0315507	5575439	18	–
	Stream	10U 0317499	5576764	21	Outlet
Roberts (Ro)	Lake	10U 0318479	5565773	21	–
	Stream	10U 0316833	5567802	21	Outlet
Swan (Sw)	Lake	09U 0562393	5613903	20	–
	Stream	09U 0561500	5614204	18	Outlet
Thiemer (Th)	Lake	09U 0642982	5598190	23	–
	Stream	09U 0642926	5599229	21	Outlet
Village Bay (Vb)	Lake^a^	10U 0343586	5560360	22	–
	Stream	10U 0343052	5560043	21	Inlet

a These sites were sampled in multiple locations because of low fish catch rates. GPS coordinate presented here is for the site where the most fish were caught. Other coordinates available from authors upon request.

### 2.2 Environmental data

We recorded environmental variables encompassed within a 1‐meter radius around each trap at each collection site. Categorical data included a list of aquatic vegetation and benthic substrates present. For each categorical variable, we generated a presence–absence matrix with as many columns as there were levels of that variable. Then, we ran a principal components analysis (PCA) on that presence–absence matrix, keeping the PC scores from a minimum of three axes (or more, if needed, to explain at least 66% of the total variance). Continuous measures include the amount of canopy cover, water depth, and flow rate (see Table 2 for details). Continuous habitat variables were scaled by *z*‐transformation. These measures were used to quantify environmental complexity, as described below.

**Table 2:** Description of environmental variables

Variable	Type	Level	Description
Flow rate	Continuous	Trap	At each trap
Depth	Continuous	Trap	At each trap
Fish caught	Continuous	Trap	Number of stickleback caught in a trap
Width	Continuous	Trap	Stream only
Substrate	Categorical	Trap	Categories: Cobbles, Gravel, Sand, Mud, Bedrock, Algal cover, Mud with Rocks, Dead Plant Matter, Clay, Rocks
Fringing habitat	Categorical	Trap	Categories: Forest, Grassy Marsh, Brushy Marsh, Muskeg, Beach, Open Water
Vegetation	Categorical	Trap	Categories: None, Emergent macrophytes (with subcategories), Submerged Macrophytes (with subcategories), Submerged Logs, Submerged Branches
Bank slope	Categorical	Trap	Categories: Vertical, Steep Sloping, Shallow, Shelf with Drop‐off, Shallow Step, Marsh (no bank)
Water clarity	Categorical	Trap	Categories:Clear, Lightly Stained, Heavily Stained
Flow modification	Categorical	Trap	Categories: Beaver Dam, Ex‐beaver Dam, Logging Detritus, Human Impounded, Channelized
Flow type	Categorical	Trap	Categories: Still, Sluggish, Laminar Fast, Turbulent Fast, Whitewater, Pool‐Riffle
Canopy coverage	Categorical	Trap	Categories: Overhead Open, Overhead Closed, Understory Closed, Understory Open, Dead Logs
Bycatch	Categorical	Trap	Categories: Trout, Salmon, Sculpin, Crayfish, Tadpoles

### 2.3 Brain dissection

To provide access to the cranium, we used scissors to make an incision just dorsal to the eye on one side of the head and cut along the outer perimeter of the eye socket to remove the eyes. The cranium was then separated from the rest of the head by cutting in a plane, anteroposteriorly, underneath the cranium, from the midline of the snout to the back of the head. The cranium was then placed in ~400 μl of formalin for additional fixation for at least 24 hrs to reduce the risk of damage to the brain during handling. We subsequently washed the cranium with isopropanol and then cut anteroposteriorly from the tip of the snout along the dorsal axis to the back of the cranium. We pinned open the sides of the cranium to expose the brain, which we removed using forceps. Fifteen to 24 undamaged brains were successfully dissected from each of the 30 populations (Table 1). Additionally, for each individual, we measured standard length (SL).

### 2.4 Imaging and measurements

We placed the still‐wet brain onto a stage micrometer and used a microscope mounted with a Canon Rebel XTi digital SLR camera to take a dorsal image of the brain at 0.75× magnification. This view captured the telencephalon, the right and left occipital lobes, and the cerebellum. An external light source at medium intensity lit the stage with minimal glare.

**Figure 1:**
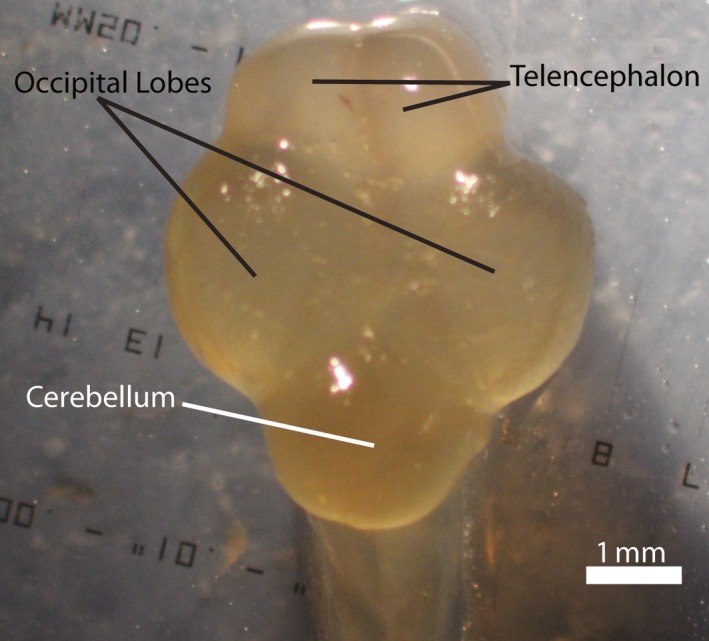
Dorsal image of a stickleback brain with the telencephalon, occipital lobes, and cerebellum indicated. The telencephalon is anterior of the occipital lobes and cerebellum. “Whole brain” was the area encompassed by tracing the outside of these regions

We used the plug‐in Object J (Vischer, 2014) in FIJI (Schindelin et al., 2012), an ImageJ‐based (Abramoff, Magalhaes, & Ram, 2004) digitization software program, to measure brain areas. For each individual, we traced the following regions: telencephalon (both lobes), occipital region (both lobes), cerebellum, and the whole brain (Figure 1). These regions were chosen because previous studies have indicated that these regions respond to environmental variation (Burns & Rodd, 2008; Burns et al., 2009; Corfield et al., 2012; Gonda et al., 2009; Kotrschal et al., 1998; Park & Bell, 2010; Pollen et al., 2007).

### 2.5 Size correction

We size corrected the brain traits using the following size‐correction formula, *M*_s,i_ = *M*_0,i_*(*L*_s_/*L*_0,i_)^*b*^, where *M*_s,i_ is the size‐corrected trait value for individual *i*, M_0,i_ is the non‐size‐corrected trait value for individual *i*,* L*_s_ is the overall mean for our log‐transformed size‐related variable across all individuals, and *L*_0,i_ is the log‐transformed size‐related variable of individual *i,* standard length in this case. (We also used head length as an alternative proxy for size; our results, not shown, were qualitatively similar.) *b* is the common, within‐group slope calculated from a linear mixed model of log10(*M*_0,i_) regressed on log10(*L*_0,i_), with watershed included as a random factor (Lleonart, Salat, & Torres, 2000; Oke et al., 2015; Reist, 1985). All analyses were conducted on size‐corrected data. This analysis and analyses described below were all conducted in RStudio, using R version 3.2.2.

### 2.6 Pooling sexes

We examined whether there was a significant sex effect, or a significant sex*population effect using a MANOVA on all the size‐corrected brain traits. Neither effect was significant. Thus, for subsequent analyses, we pooled sexes. We used MANCOVA as well to test whether sexes differ in their habitat use, with environmental variables as dependent variables, and sex, standard length, and population as independent variables.

### 2.7 Testing the CFH

Before testing the CFH, we first tested whether brain (and brain subregion) sizes actually differ among populations. We used MANOVA (package stats:*manova*) on all four brain traits (total, telencephalon, occipital lobes, and cerebellum sizes) and type II ANOVA on each brain trait individually (package car:*Anova* “type = II” on the object output from package stats:*lm*) in R. Here and throughout, we tested brain traits individually, rather than taking a PCA approach, to maintain interpretability of any findings, as there may be different CFH interpretations for different brain regions. After finding that there were indeed brain size differences among populations (Table 3), we set out to test whether they might be explained by the CFH.

**Table 3:** Significant Habitat, Watershed, and Interaction effects on relative brain size and relative brain region size. Results of type II ANOVAs reported for individual regions, as well as MANOVA of all four brain traits. η^2^, in bold, is the percent variance explained by each term in the model (R:BaylorPsychEd:*EtaSq*). Sample sizes for each population in Table 1

Brain region	Habitat (SumSq/*df*/*F*/*p*/η^2^)	Watershed (SumSq/*df*/*F*/*p*/η^2^)	Hab*Wshd Interaction (SumSq/*df*/*F*/*p*/η^2^)
Total brain	0.21/1/17.7/.00/**.01**	5.45/14/32.3/.00/**.38**	2.09/14/12.3/.00/**.14**
Telencephalon	0.21/1/7.7/.01/**.01**	6.41/14/16.4/.00/**.29**	0.91/14/2.33/.00/**.04**
Cerebellum	0.22/1/15.7/.00/**.01**	4.86/14/25.2/.00/**.34**	1.70/14/8.8/.00/**.12**
Occipital	0.13/1/12.79/.00/**.01**	2.97/14/21.1/.00/**.30**	1.40/14/9.9/.00/**.14**
MANOVA	*F*_4,516_ = 5.4; *p* = .00 η^2^ = .01	*F*_56,2076_ = 13.3; *p* = .00 η^2^ = .29	*F*_56,2076_ = 8.5; *p* = .00 η^2^ = .04

Intuitively we expected that lakes would be simpler habitats than streams, because the latter involve greater heterogeneity in flow regimes and often entail meandering channels through broad marshes. We tested whether there was a habitat (i.e., lake vs. stream) effect on overall brain morphology using MANOVA, and each brain trait individually using type II ANOVA, all on individual level data. (We retained watershed—that is, the unique watershed in which each lake–stream pair was found—as a variable in our model to account for the paired nature of our lake–stream samples.) Lake‐versus‐stream categorization might not cleanly correspond to environmental heterogeneity, however, because there is appreciable intra‐habitat‐type variation in environmental characteristics (Stuart et al., *in revision*). Therefore, to test the CFH more concretely, we quantified environmental complexity directly at each site, using two approaches.

### 2.8 Multivariate measure of environmental complexity: Euclidean distance to local environmental centroid

First, we calculated the Euclidean distance of each trap's environmental data to the environmental centroid of all traps at that trap's collection site. Sites with greater environmental complexity should exhibit greater mean Euclidian distances (greater variance among traps). We used ANOVA at the level of trap to test whether there are indeed significant differences among sites in this measure of habitat complexity, and, again at the trap level, used ANCOVA to test for habitat and watershed effects on this complexity metric.

We then tested whether complexity influences brain size, per the CFH. We calculated the mean trap‐to‐centroid Euclidean distances for each population and then used MANCOVA to test whether population‐mean brain morphology covaries with population‐mean, environmental Euclidean distance. (Again, we kept watershed in this model.) We then used type II ANOVAs, with watershed as a factor, to investigate the relationship between population means for individual brain regions and population means for Euclidean complexity.

### 2.9 Univariate measures of environmental complexity: Environmental trait standard deviations

Second, we calculated each environmental variable's standard deviation at each site. Habitats with high standard deviations should be more complex than those whose environmental variables have little variation around their mean. We then used MANCOVAs testing whether overall brain morphology covaries with the standard deviation of each environmental variable, including watershed as a factor. To further explore the effect of individual environmental variables on individual brain traits, we ran type II ANOVAs pairwise for each environmental trait's standard deviation effect on each brain subregion area with watershed as a factor.

We note that both of our measures of environmental complexity assume that complexity is environmental variation across space; that is, we use microspatial environmental variation as our proxy for complexity.

## 3 Results

### 3.1 Habitat effect on brain size

**Figure 2:**
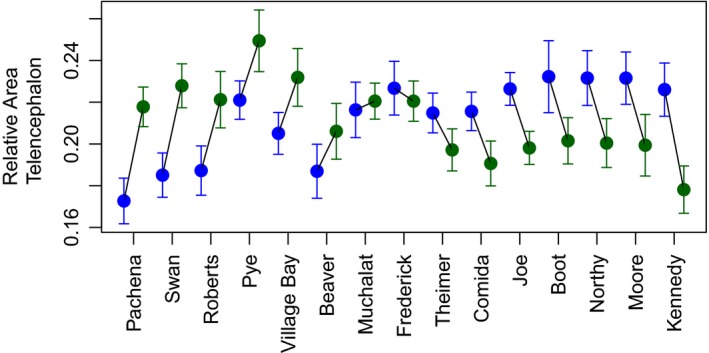
Population mean (±1*SE*), size‐corrected area of the telencephalon. Lines connect lake (blue) to stream (green) means for each pair. Pairs are ordered from largest to smallest stream‐to‐lake difference. Plots for other brain regions are qualitatively similar, showing similar habitat, watershed, and interaction effects

Overall, brain (and brain subregion) sizes varied significantly among the 30 populations (Table 3). Per the CFH, we expected that lake fish would tend to have smaller brain sizes than stream fish, as lakes should be simpler than streams in environmental complexity. There was indeed a habitat effect on brain size, but it was small, with an effect size approximately an order of magnitude smaller than the effects of both watershed and a habitat*watershed interaction (Table 3). The significant watershed*habitat interaction indicates that the direction of lake–stream divergence in brain size is inconsistent among watersheds (Table 3; Figure 2). In telencephalon, for example, a lake population had larger telencephala than its corresponding stream population in eight of 15 watersheds, while streams had larger telencephala in seven of 15 watersheds (Figure 2). Similar trends hold true for the other brain regions (Table 4). Moreover, this interaction effect is due to both significant among‐lake variation in brain anatomy and significant among‐stream variation (Table 5). Thus, the a priori expectation for a lake‐versus‐stream relationship with the CFH is not met when considering habitat categories alone. We next tested whether brain morphology varies with quantitative estimates of habitat complexity.

**Table 4:** Effect of habitat on relative brain (and relative brain region) size for each watershed. L>S indicates that the lake population has larger values than its adjoining stream population; S>L vice versa

Watershed	Total brain		Telencephalon		Cerebellum		Occipital	
Beaver		S > L		S > L		S > L		S > L
Boot		S > L*		S > L		S > L		S > L*
Comida	L > S		L > S*		L > S*		L > S	
Frederick	L > S**		L > S			S > L**	L > S*	
Joe		S > L***		S > L		S > L***		S > L
Kennedy		S > L	L > S		L > S**			S > L
Moore		S > L	L > S			S > L**		S > L*
Muchalat	L > S***		L > S*		L > S*		L > S***	
Northy		S > L*		S > L		S > L*		S > L*
Pachena	L > S***		L > S**		L > S***		L > S***	
Pye	L > S***		L > S*		L > S***		L > S	
Roberts	L > S*		L > S		L > S		L > S*	
Swan	L > S***		L > S		L > S***		L > S***	
Thiemer		S > L**		S > L		S > L		S > L
Village Bay	L > S		L > S		L > S*		L > S	

Significance indicated by asterisks: *p ≤ .05; **p < .01; ***p < .001. Justification differences serve as a visual aid. Sample sizes for each population in Table 1.

**Table 5:** Both among‐lake and among‐stream variation in brain size contribute to the habitat*watershed interaction. Lake(L)/stream(S) output from MANOVA or type II ANOVA of brain region for lake populations only and stream populations only

Brain region	Sum Sq. (L/S)	*df* (L/S)	*F* (L/S)	*p* (L/S)
Whole Brain	5.4/2.1	14/14	35.8/11.4	.00/.00
Telencephalon	4.5/2.9	14/14	11.5/7.3	.00/.00
Cerebellum	5.3/1.3	14/14	30.8/6.0	.00/.00
Occipital	3.0/1.4	14/14	23.9/8.7	.00/.00
MANOVA	NA	14/14	12.7/6.7	.00/.00

### 3.2 Multivariate: Euclidean distance to local environmental centroid

An ANOVA reveals among‐population variation in environmental complexity, measured as the Euclidean distance to the centroid of the local environmental data (*F*_29_ = 15.0, *p* < .001). Moreover, there are significant effects (all *p* < .001) of watershed (proportion variance explained: η^2^ = .07), habitat (η^2^ = .007, suggesting that streams are less complex than lakes), and a habitat*watershed interaction (η^2^ = .11) on this measure of environmental complexity. Thus, lake and stream populations vary in their environmental complexity which, according to the CFH, should drive the evolution of stickleback brain morphology. However, the interaction effect suggests that habitat per se (lake vs. stream) may be less important than quantitative measures of habitat complexity.

We used MANCOVA (and type II ANOVAs) to test whether multivariate (or univariate) brain size measures indeed covary positively with this measure of environmental complexity. We found no significant covariation between spatial environmental complexity (mean Euclidean distance from the centroid of the local environmental data) and overall brain morphology, nor the mean of any of the univariate brain traits (Table 6).

**Table 6:** Complexity measured as population‐mean Euclidean distance to the environmental centroid provides minimal evidence for the CFH. Based on type II ANOVAs or MANCOVA

Brain region	Complexity (SumSq/*df*/*F*/*p*)	Watershed (SumSq/*df*/*F*/*p*)
Total brain	0.00/1/0.5/.50	0.29/14/2.4/.05
Telencephalon	0.01/1/2.3/.15	0.35/14/6.3/.00
Cerebellum	0.01/1/1.3/.28	0.27/14/2.9/.03
Occipital	0.00/1/0.6/.46	0.16/14/2.0/.10
MANOVA	*F*_4_ = 0.05; *p* = .99	*F*_56_ = 1.63; *p* = .04

### 3.3 Univariate: Environmental trait standard deviations

We also used the standard deviation of each univariate environmental variable, by site, as a proxy for environmental complexity. MANOVA revealed significant relationships between the standard deviation of some environmental variables and population‐mean brain morphology (Table 7). Type II ANOVAs also revealed several significant brain size–environmental complexity relationships (Table 7). The most striking were consistent, positive relationships between our brain traits and complexity in lake and stream bottom substrate (we also found several negative relationships with other environmental variables). This ANOVA approach generated many tests of the same hypothesis (four brain regions by 44 environmental traits = 176 tests). However, a *Z*‐transform test for combining *p*‐values (Whitlock, 2005) rejects the common null hypothesis that there is no relationship between complexity and brain traits (*Z*_S_ = 13.5, *p* < .0001). Thus, at least some of the significant ANOVA relationships in Table 7 are real. A false discovery rate (fdr) analysis (package *qvalue:qvalue*) found four comparisons that were still significant at an fdr level of .05: Both substrate PC 4 and substrate PC6 significantly explained variation for both relative cerebellum size and relative whole brain size (Table 7).

**Table 7:** Results of MANOVA and ANOVA tests for by‐site brain region means against by‐site standard deviations for each environmental variable, with watershed as a factor. Percent variance explained refers to the amount of variance explained by a PC axis, where PCA was conducted separately for each environmental variable

Environmental variable (% var. explained)	MANOVA	Total brain	Telencephalon	Cerebellum	Occipital
substrate.pc1 (21%)	–	–	–	–	–
substrate.pc2 (12%)	–	*	*	**	–
substrate.pc3 (10%)	–	–	–	*	–
substrate.pc4 (10%)	*	** a	*	***,^a^	*
substrate.pc6 (8%)	–	**,^a^	*	***,^a^	**
fringe.habitat.pc1 (29%)	–	–	–	*	–
fringe.habitat.pc3 (17%)	***	–	–	*	–
vegetation.pc6 (5%)	–	–	–	*	–
vegetation.pc10 (4%)	*	–	–	–	–
bank.pc2 (15%)	–	–	(*)	–	–
bank.pc3 (15%)	**	–	(**)	–	–
water.clarity.pc1 (52%)	*	–	–	–	–
flow.category.pc1 (32%)	–	–	–	(*)	–
flow.category.pc2 (21%)	*	–	–	–	–
canopy.pc2 (15%)	–	(*)	(*)	–	(*)
canopy.pc5 (13%)	*	–	–	–	–
bycatch.pc3 (17%)	*	–	–	–	–

*<.05; *^*^<.01; *^**^<.001.

() denotes negative relationship revealed by ANOVA. Variables for which no test was significant are: flow rate; depth; substrate PC5; fringe habitat PCs 2,4; vegetation PCs 1–5, 7–9, 11–13; bank PCs 1,4; water clarity PCs 2,3; flow category PC 3; canopy PCs 1,3,4; bycatch PCs 1,2,4.

a Univariate comparisons that were still significant with a false discovery rate at the .05 level.

## 4 Discussion

The Clever Foraging Hypothesis predicts a positive relationship between habitat complexity and brain size (Huntigford & Wright, 1992; Kotrschal et al., 1998; Park & Bell, 2010), particularly for the telencephalon (Bauchot et al., 1977; Broglio et al., 2003, 2005; Gonda et al., 2009; Park & Bell, 2010; Rodriguez et al., 2002) but also for other brain regions (Bauchot et al., 1977; Kotrschal et al., 1998). We surveyed stickleback in 30 populations—15 lake–stream pairs—for this relationship.

Although there were differences among watersheds in standard length‐corrected brain size (including total brain size and the relative size of brain subregions; Table 3), we found divergence in brain morphology between lakes and streams to be inconsistent (i.e., a weak habitat effect and a strong habitat*watershed interaction effect; Table 3). Assuming that lakes are generally less environmentally complex than streams, this result provides little support for the CFH. However, there is appreciable intrahabitat environmental variation (i.e., among lakes and among streams; Stuart et al. *in revision*), and simple lake–stream categorization might fail to capture environmental heterogeneity important to brain evolution (Park & Bell, 2010).

We thus quantified two continuous metrics of habitat complexity to test for a relationship between complexity and brain morphology. The first metric—Euclidean distance, by site, of each trap's environmental makeup to that site's centroid in environmental space—revealed significant among‐site differences in multivariate habitat complexity, but no relationship between habitat complexity and overall brain morphology nor the morphology of brain subregions (Table 6).

Our univariate measures of habitat complexity (the standard deviation around the mean score for each environmental variable, by site) did reveal a few environmental characteristics that were positively correlated with brain size (and some that were negatively correlated). A positive correlation between the relative size of the whole brain as well as the cerebellum and complexity in lake–stream bottom substrate stands out in particular. Threespine stickleback commonly forage on the substrate in these lakes and streams (Snowberg, Hendrix, & Bolnick, 2015). Along a benthic–limnetic habitat axis, more benthic stickleback have been shown to be better at navigating a maze to find a food reward (Odling‐Smee et al., 2008), perhaps indicative of better spatial memory (Girvan & Braithwaite, 1998). So it may be that larger brains are required to detect and pursue prey in more complex, benthic substrates. However, vegetation structure revealed no relationship with brain morphology, despite what could have been a similar a priori expectation.

It is interesting to note that we found no differences in brain morphology between sexes, although MANCOVA revealed significant differences in habitat use between the sexes (*p* < .001; population [*p* < .001] and standard length [*p* = .003] were also significant predictors of environmental variation). Assuming that different habitat use reflects different habitat complexity (and that our trap environmental data are representative of total complexity experienced), that we found no brain differences among the sexes is further evidence against the CFH. We also note that by collecting fish during only a few weeks in the summer, we may be missing temporal variation in habitat use and environmental conditions, and therefore missing important variation in complexity that may explain some of the variation we see in brain size. Moreover, our measurements of habitat complexity are coarse and we have no way of knowing what really represents complexity from a stickleback's perspective. We hope our data provide a good starting point for future research.

On the whole, support for the Clever Foraging Hypothesis is weak in our system, despite our large power to detect any patterns (30 populations from 15 watersheds, 606 fish). Why might this be? First, we measured brain size but not shape. If the shape of different brain regions is more indicative of neurological complexity than size, then we may not have quantified the appropriate evolutionary outcome predicted by the CFH (Burns & Rodd, 2008; Burns et al., 2009; Park & Bell, 2010). Indeed, Park and Bell (2010) found benthic–limnetic differences in telencephalon convexity consistent with the CFH. Moreover, structures within each subregion may evolve independently in response to selection pressures (Barton & Harvey, 2000; Corfield et al., 2012) from complex habitats and our gross anatomical approach would not capture that. That said, we did find relatively strong correlations between brain size and functional morphological traits (Data S1), suggesting that even our gross measurements reflect ecomorphologically relevant variation. Regardless, future studies investigating the CFH in the lake–stream stickleback system would benefit from a geometric morphometric analysis of the brain, preferably on fresh specimens not subject to potential preservation effects. The field would also benefit from a better understanding of how well stickleback brain size (and shape) correlate with neurological capacity, as it is neurological capacity that is really under selection. Second, other selective demands may be more important in shaping the brain and its underlying neurological capacity. In particular, stickleback are a highly social species, and brain size may be modulated more by social interactions than differences in the environment. Thus, although we were pioneering in measuring multiple aspects of lake and stream environments, it is possible that adding data about the behavioral ecology of this system would better explain differences among watersheds in brain morphology.

## Conflict of interest

None declared.
