# Supplementary material for: Brain morphology of the threespine stickleback (Gasterosteus aculeatus) varies inconsistently with respect to habitat complexity: A test of the Clever Foraging Hypothesis
Source: Ecol Evol. 2017 Apr 4;7(10):3372–80. doi: 10.1002/ece3.2918 (PMC5433989; doi:10.1002/ece3.2918)
Supplement: Supplementary file 1 [file ECE3-7-3372-s001.docx]

Supplementary Data: Brain by Functional Morphology Relationships

Here, we present relationships between brain traits and two functional morphological data subsets—trophic morphology and swimming morphology—to provide context to other morphological characters. The functional morphological datasets are taken from another manuscript (Stuart et al., Contrasting effects of environment and genetics generate a predictable continuum of parallel evolution, *Nature Ecology and Evolution,* in Revisions).

We tested for associations between each pairwise combination of brain trait (i.e., whole brain area, telencephalon area, etc.) and functional morphological trait (e.g., gill raker number, gape width, fin area, etc.; described in the *Nature E&E* manuscript, and in Supplementary Data Table 1, below). To accomplish this goal, for each pairwise phenotype/environment trait combination, we first made a full mixed-linear model with the R package *lme4*: lmer(phenotypic trait ~ environmental trait + habitat +(1|pair). We then used ANOVA to compare to this full model to a reduced model without the environmental variable: lmer(phenotypic trait ~ habitat + (1|pair). We retained the t-values from the full linear models and consider them proxies for the relationships between brain and functional traits.

We found numerous significant correlations between brain traits and functional traits. Using a permissive P-value cutoff of 0.05, we observed an excess of apparently significant comparisons, suggesting that there are strong correlations between brain and functional traits. Below, we present heat maps showing the strength (t-statistic) of many trait-environment correlations. In each figure, red shading corresponds to large t-values that show that a functional trait is positively correlated with the focal brain phenotype for any given cell. Blue corresponds to larger negative t-values that show that the functional trait is negatively correlated with a given phenotype. White corresponds to t-values near zero. However, all t-values plotted below are significantly different from zero; that is, for readability, in the following figures we only plot t-values for brain-function correlations that are significantly different from zero. Traits that were not significantly associated with any brain variable (or vice versa) were dropped from the heatmaps for ease of presentation. The dendrogram shows the distance between variables. There are two heat maps, described below.

A) We tested the relationships between our four brain traits and 17 morphological traits we deem *a priori* to likely be important for trophic function. The largest t-statistic in this plot (i.e., most red) was 3.5, and the smallest (i.e., most blue) was -7.5.


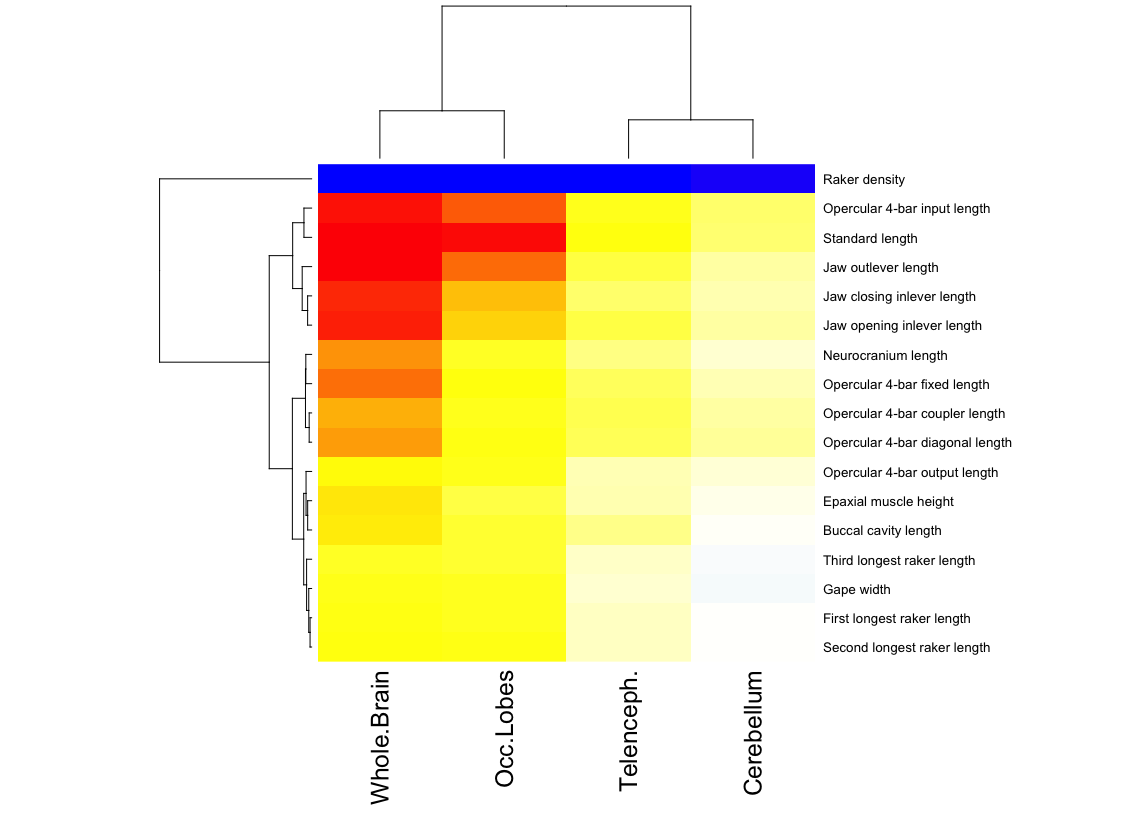


Occipital Lobe Area

Telencephalon Area

Cerebellum Area

Whole Brain Area

B) We tested the relationships between our four brain traits and 15 morphological traits we deem *a priori* to likely be important for swimming function. The largest t-statistic in this plot (i.e., most red) was 8.5, and the smallest (i.e., most blue) was -8.5.


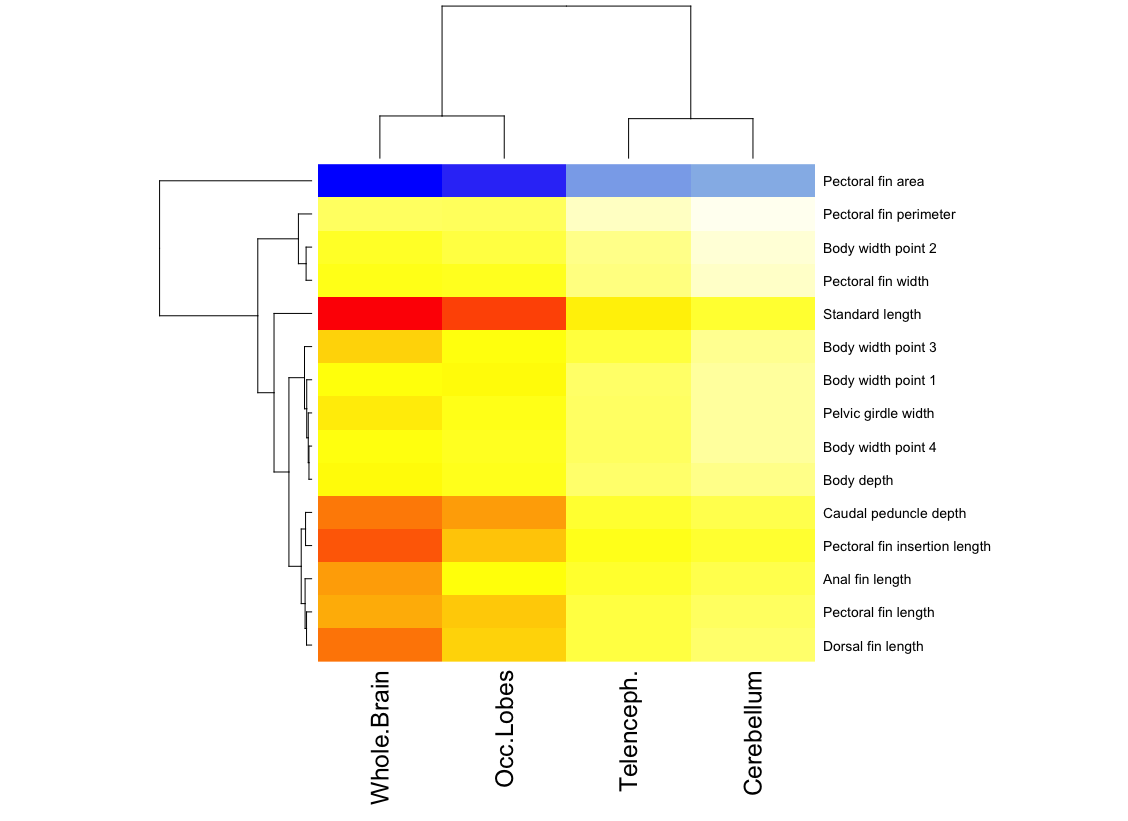
**Supplementary Table 1**. Description of linear morphological measurements important for functional morphology. Lengths were taken from digital images.

Cerebellum Area

Telencephalon Area

Occipital Lobe Area

Whole Brain Area

| Trait | Method | Description |
| --- | --- | --- |
| Dorsal fin length^b^ | Lateral image | Length, at insertion points |
| Caudal peduncle depth^b^ | Lateral image | Depth, narrowest point of caudal peduncle |
| Anal fin length^b^ | Lateral image | Length, at insertion points |
| Pectoral fin insertion length^b^ | Lateral image | Length, dorsal to ventral insertion |
| Body depth^b^ | Lateral image | Depth, anterior insertion of first dorsal spine to anterior point of pelvic girdle |
| Pectoral fin width^b^ | Lateral image | Width, measured from distal ends of outer fin rays. |
| Pectoral fin length^b^ | Lateral image | Length, longest fin ray |
| Pectoral fin perimeter^b^ | Lateral image | Perimeter |
| Pectoral fin area^b^ | Lateral image | Area |
| Standard length^a,b^ | Calipers | Length, tip of snout to end of caudal peduncle |
| Buccal cavity length^a^ | Ventral image | Length, tip of snout to anterior point of ectocoracoid |
| Gape width^a^ | Ventral image | Width, distance between mouth corners |
| Body width pt.1^b^ | Ventral image | Width, jawbone to jawbone, centered at the eyes |
| Body width pt.2^b^ | Ventral image | Width, distance between posterior points of ectocoracoid |
| Pelvic girdle width^b^ | Ventral image | Width, between insertions of pelvic spines |
| Body width pt.3^b^ | Ventral image | Width, across anterior insertion of anal fin |
| Body width pt.4^b^ | Ventral image | Width, across posterior insertion of anal fin |
| Right side gill raker number^a^ | Count | Counted *in situ*, under a microscope |
| First longest raker length^a^ | Raker image | Dissected raker, measured from photograph. |
| Second longest raker length^a^ | Raker image | Dissected raker, measured from photograph |
| Third longest raker length^a^ | Raker image | Dissected raker, measured from photograph |
| Raker density^a^ | Raker image | Dissected raker, length spanned by five longest rakers |
| Jaw opening inlever length^a^ | Jaw image | Dissected lower jaw, pts. 8 -> 9 Fig. 2 ^1^; only 20 fish per site |
| Jaw closing inlever length^a^ | Jaw image | Pt. 8 (Fig. 2^1^ ) -> dorsalmost point of Articular bone; only 20 fish per site |
| Jaw outlever length^a^ | Jaw image | Pts. 2 -> 8 of Fig. 2 in^1^; only 20 fish per site |
| Epaxial muscle height^a^ | Lateral image | Pts. 3 -> 4 of Fig. 2 in^1^, only 20 fish per site |
| Epaxial muscle cross sectional area^a^ | Epaxial image | Severed head at base of skull, following line between pts. 3 & 4 of Fig. 2 in^1^; photographed and measured epaxial area; only 20 fish per site |
| Neurocranium length^a^ | Lateral image | Pts. 1 -> 3 of Fig. 2 in {McGee:2013tx}; only 20 fish per site |
| Opercular 4-bar fixed length^a^ | Lateral image | Pts. 8 -> 10 of Fig. 2 in^1^; only 20 fish per site |
| Opercular 4-bar coupler length^a^ | Lateral image | Pts. 9 -> 11 of Fig. 2 in^1^; only 20 fish per site |
| Opercular 4-bar input length^a^ | Lateral image | Pts. 10 -> 11 of Fig. 2 in^1^; only 20 fish per site |
| Opercular 4-bar output length^a^ | Lateral image | Pts. 8 -> 9 of Fig. 2 in^1^; only 20 fish per site |
| Opercular 4-bar diagonal length^a^ | Lateral image | Pts. of Fig. 2 in^1^; only 20 fish per site  9 -> 10 |

^a^Trophic Trait Subset. ^b^Swimming Trait Subset.

Supplementary References

1. McGee, M. D. & Schluter, D. Functional basis of ecological divergence in sympatric stickleback. *BMC Evolutionary Biology* **13,** 277 (2013).
